# Protease Activity of *Campylobacter jejuni* HtrA Modulates Distinct Intestinal and Systemic Immune Responses in Infected Secondary Abiotic IL-10 Deficient Mice

**DOI:** 10.3389/fcimb.2019.00079

**Published:** 2019-03-29

**Authors:** Anna-Maria Schmidt, Ulrike Escher, Soraya Mousavi, Manja Boehm, Steffen Backert, Stefan Bereswill, Markus M. Heimesaat

**Affiliations:** ^1^Institute of Microbiology, Infectious Diseases and Immunology, Charité - University Medicine Berlin, Corporate Member of Freie Universität Berlin, Humboldt-Universität zu Berlin, Berlin Institute of Health, Berlin, Germany; ^2^Department of Biology, Institute for Microbiology, Friedrich Alexander University Erlangen-Nuremberg, Erlangen, Germany

**Keywords:** *Campylobacter jejuni*, secondary abiotic IL-10^−/−^ mice, serine protease activity, high-temperature requirement A (HtrA), host-pathogen-interaction, intestinal immunopathology, extra-intestinal and systemic immune responses, bacterial translocation

## Abstract

Even though human *Campylobacter jejuni* infections are progressively increasing worldwide, the underlying molecular mechanisms of pathogen-host-interactions are still not fully understood. We have recently shown that the secreted serine protease HtrA plays a key role in *C. jejuni* cellular invasion and transepithelial migration *in vitro*, and is involved in the onset of intestinal pathology in murine infection models *in vivo*. In the present study, we investigated whether the protease activity of HtrA had an impact in *C. jejuni* induced acute enterocolitis. For this purpose, we perorally infected secondary abiotic IL-10^−/−^ mice with wildtype *C. jejuni* strain NCTC11168 (11168^WT^) or isogenic bacteria carrying protease-inactive HtrA with a single point mutation at S197A in the active center (11168^HtrA−S197A^). Irrespective of the applied pathogenic strain, mice harbored similar *C. jejuni* loads in their feces and exhibited comparably severe macroscopic signs of acute enterocolitis at day 6 postinfection (p.i.). Interestingly, the 11168^HtrA−S197A^ infected mice displayed less pronounced colonic apoptosis and immune cell responses, but enhanced epithelial proliferation as compared to the 11168^WT^ strain infected controls. Furthermore, less distinct microscopic sequelae in 11168^HtrA−S197A^ as compared to parental strain infected mice were accompanied by less distinct colonic secretion of pro-inflammatory cytokines such as MCP-1, IL-6, TNF, and IFN-γ in the former as compared to the latter. Strikingly, the S197A point mutation was additionally associated with less pronounced systemic pro-inflammatory immune responses as assessed in serum samples. In conclusion, HtrA is a remarkable novel virulence determinant of *C. jejuni*, whose protease activity is not required for intestinal colonization and establishment of disease, but aggravates campylobacteriosis by triggering apoptosis and pro-inflammatory immune responses.

## Introduction

*Campylobacter jejuni* is a Gram-negative, spiral-shaped, flagellated bacterium that grows under microaerobic conditions at a temperature optimum of 37–42°C (Parkhill et al., 2000; Brøndsted et al., 2005; Young et al., 2007). *C. jejuni* is a commensal in the gastrointestinal tract of various animal hosts such as swine, cattle, and birds (Fouts et al., 2005). Consequently, the consumption of contaminated meat products, raw milk and water are the most frequent sources of *C. jejuni* infections in humans (Bereswill et al., 2011; Pielsticker et al., 2012). The infection comes along with abdominal pain, fever, myalgia, and watery or bloody diarrhea. In the acute stage of human campylobacteriosis, manifestations like crypt abscesses, ulcerations, and accumulation of immune cells in the colon can be observed (Kist and Bereswill, 2001; Backert et al., 2017). In addition, post-infectious complications such as Guillain–Barré syndrome and Miller Fisher syndrome can emerge in a minority of patients (Masanta et al., 2013; Goodfellow and Willison, 2016; Backert et al., 2017). *C. jejuni* bacteria enter the host intestine *via* the oral route and colonize the distal ileum and colon by attaching to epithelial cells (Kist and Bereswill, 2001; Boehm et al., 2018). To reach deeper tissues and cause inflammatory responses during the infection process, *C. jejuni* need to cross the epithelial barrier, which is accomplished by paracellular transmigration as well as invasion into intestinal epithelial cells (Boehm et al., 2012). After crossing the intestinal epithelial barrier and invasion of the underlying submucosa, *C. jejuni* can enter the mesenteric lymph nodes (MLN), and may reach different organs such as the spleen or liver via the bloodstream, thereby causing extra-intestinal inflammation (Backert et al., 2013).

The exact mechanisms how *C. jejuni* induce acute and invasive enterocolitis remain yet unclear. However, it has been observed that *C. jejuni* interferes with several signal transduction pathways of the innate immune system. We have recently shown that the pattern recognition receptors Toll-like receptor-4 (TLR-4) (Bereswill et al., 2011; Haag et al., 2012a; Otto et al., 2012) and nucleotide-oligomerization-domain-2 (Nod2) (Heimesaat et al., 2017a,b) play a pivotal role in the development of immunopathology during *C. jejuni* infection. Moreover, *C. jejuni* rapidly activates MAPK/ERK (mitogen-activated protein kinases/extracellular signal-regulated kinases) as well as the pro-inflammatory transcription factor NF-κB (nuclear factor kappa-light-chain-enhancer of activated B cells) that up-regulates the transcription of a variety of immune response genes (Chen et al., 2006; Hu et al., 2006). It has been shown that *C. jejuni* can enhance the production of interleukin (IL)−8, a chemoattractant that recruits dendritic cells, macrophages and neutrophils to the site of infection (Jagusztyn-Krynicka et al., 2009). Internalization of *C. jejuni* by dendritic cells results in their activation and triggers adaptive immune response. This leads to an enhanced expression of a variety of pro-inflammatory cytokines, namely IL-1β, IL-6, IL-8, IL-10, IL-12, Interferon (IFN) -γ and tumor necrosis factor (TNF) in immune cells (Hu et al., 2006; Jagusztyn-Krynicka et al., 2009).

*C. jejuni* is known to express numerous virulence factors for its adhesion to, invasion into and transmigration across gut epithelial cells (Masanta et al., 2013). Recent studies suggest that high-temperature requirement A (HtrA) has a major impact on these processes and thereby increases virulence of several pathogens (Boehm et al., 2012, 2018). So far, HtrA proteases and their orthologs have been investigated in bacterial pathogens such as *Escherichia coli* (Jiang et al., 2008; Krojer et al., 2008), *Helicobacter pylori* (Hoy et al., 2010; Albrecht et al., 2018), *Legionella pneumophila* (Schubert et al., 2015), *Borrelia burgdorferi* (Coleman et al., 2013), *Bacillus anthracis* (Chitlaru et al., 2011, 2017), *Streptococcus pneumoniae* (Cassone et al., 2012) or defined *Chlamydia* species (Wu et al., 2011). Various studies revealed distinct bacterial virulence features of HtrA orthologs. For example, inactivation of HtrA in *Clostridium difficile* resulted in more pronounced virulence as shown in a hamster model (Bakker et al., 2014). In contrast, overexpression of HtrA in *Streptococcus pneumoniae* was associated with enhanced virulence of the pathogen *in vitro*, whereas the inactivation of HtrA resulted in attenuated pneumonia in infected mice (de Stoppelaar et al., 2013).

HtrA constitutes a serine protease with an additional chaperone function, which provides a protective effect against temperature- and oxidative stress induced protein degradation in the periplasm (Krojer et al., 2008; Boehm et al., 2018). The protein consists of a signal peptide, a trypsin-like serine protease module and one or two PDZ (post synaptic density protein (PSD59), Drosophila disc large tumor suppressor (Dlg1), and zonula occludens-1 protein (ZO-1)) domains that enable protein-protein interactions (Brøndsted et al., 2005; Backert et al., 2018). Surprisingly, HtrA is not only acting intracellularly within the bacteria, but is also actively secreted into the extracellular environment, where it can degrade extracellular host proteins like the cell surface adhesion protein and tumor-suppressor E-cadherin (Boehm et al., 2012, 2013 Hoy et al., 2012; Backert et al., 2013; Heimesaat et al., 2014a). Cleavage of the extracellular E-cadherin domain leads to the depletion of adherence junctions. The following downregulation of barrier functions enables *C. jejuni* to transmigrate across the polarized epithelium (Boehm et al., 2012; Hoy et al., 2012). *In vitro* experiments revealed that deletion of the *htrA* gene or expression of a HtrA protease activity-deficient point mutant variant (serine residue 197 replaced by alanine) in *C. jejuni* leads to significantly reduced E-cadherin cleavage and transmigration across polarized epithelial cells, but did not cause loss of bacterial motility (Boehm et al., 2013). The finding that the S197A point mutation abolished protease activity, but did not affect HtrA secretion, led to the assumption that HtrA secretion does not require proteolytic activity (Boehm et al., 2013). Recent studies in the secondary abiotic IL-10^−/−^ mouse model infected with a *C. jejuni htrA* deletion mutant (11168^Δ*htrA*^) showed notably less severe clinical symptoms and reduced intestinal as well as extra-intestinal immunopathological sequelae of infection as compared to 11168^WT^ strain infected control mice (Heimesaat et al., 2014a). The role of HtrA during *C. jejuni* induced immunopathology was further confirmed in conventional infant mice which showed reduced intestinal and extra-intestinal (liver, kidney and lung) immune responses after infection with the 11168^Δ*htrA*^ mutant in comparison to 11168^WT^ infection (Heimesaat et al., 2014b). However, the role of HtrA's protease activity in *C. jejuni* infected mice was not yet investigated.

For a long time, investigations of distinct *C. jejuni* pathogenicity factors *in vivo* have been hampered by the scarcity of appropriate models (Masanta et al., 2013; Heimesaat and Bereswill, 2015). Conventional mice, for instance, are well protected from *C. jejuni* infection even upon peroral challenge with high pathogenic loads due to the physiological colonization resistance exerted by the murine commensal gut microbiota (Bereswill et al., 2011; Fiebiger et al., 2016). Following depletion of the intestinal microbiota by broad-spectrum antibiotic treatment, however, secondary abitoic mice can be stably infected with *C. jejuni* following peroral infection, but do not display gross pathogen induced symptoms seen in human campylobacteriosis (Bereswill et al., 2011). Given that mice are approximately 1,000 times more resistant to TLR-4 ligands including lipopolysaccharide and lipooligosacchariade and IL-10 gene deficiency renders mice susceptible to TLR-4 ligands (Warren et al., 2010; Haag et al., 2012a), we could recently show that upon depletion of the gut microbiota by broad-spectrum antibiotic treatment, IL-10^−/−^ mice could not only be stably infected with high *C. jejuni* loads, but also developed acute, non-self-limiting *C. jejuni* induced enterocolitis within 1 week postinfection (p.i.), thereby mimicking key features of severe campylobacteriosis in immunocompromized patients (Haag et al., 2012a; Heimesaat et al., 2014c, 2017a).

In order to investigate the role of *C. jejuni* HtrA protease activity *in vivo*, we here infected secondary abiotic IL-10^−/−^ mice with the *C. jejuni* strain 11168^HtrA−S197A^ which expresses the protease activity-deficient point mutant variant and used the corresponding 11168^WT^ as reference. We addressed bacterial colonization efficiencies and translocation rates to extra-intestinal tissue sites and surveyed clinical symptoms as well as intestinal and extra-intestinal including systemic inflammatory immune responses following peroral infection with respective strains.

## Materials and Methods

### Generation of Secondary Abiotic Mice

Female and male IL-10^−/−^ mice (all in C57BL/6j background) were bred and maintained under specific pathogen free (SPF) conditions in the same unit of the Forschungseinrichtungen für Experimentelle Medizin (FEM, Charité - University Medicine Berlin). To order to assure stable gastrointestinal *C. jejuni* colonization and override physiological colonization resistance (Bereswill et al., 2011), secondary abiotic (i.e., gnotobiotic) mice virtually lacking an intestinal microbiota were generated (Heimesaat et al., 2006; Bereswill et al., 2011; Haag et al., 2012a). In brief, immediately post weaning 3 weeks old mice were subjected to a 10-week course of broad-spectrum antibiotic treatment by adding ampicillin plus sulbactam (1 g/L; Ratiopharm, Germany), vancomycin (500 mg/L; Cell Pharm, Germany), ciprofloxacin (200 mg/L; Bayer Vital, Germany), imipenem (250 mg/L; MSD, Germany) and metronidazole (1 g/L; Fresenius, Germany) to the autoclaved drinking water (*ad libitum*). To assure antibiotic washout, the antibiotic cocktail was replaced by autoclaved tap water 2 days prior infection.

### *C. jejuni* Infection

Mice (3 months of age) were then perorally infected with 10^9^ colony forming units (CFU) of the *C. jejuni* parental strain NCTC11168 (11168^WT^) or the isogenic mutant strain 11168^HtrA−S197A^ of the serine protease gene *htrA* (Bæk et al., 2011) in a volume of 0.3 mL phosphate buffered saline (PBS; Gibco, life technologies, UK) on two consecutive days (days 0 and 1) by gavage as reported previously (Bereswill et al., 2011). Animals were continuously maintained in a sterile environment (autoclaved food and drinking water or sterile antibiotic cocktail) and handled under strict aseptic conditions to prevent from contaminations.

### Clinical Conditions

Before and after *C. jejuni* infection the clinical conditions of mice were assessed on a daily basis by using a standardized cumulative clinical score (maximum 12 points), addressing the abundance of blood in feces (0: no blood; 2: microscopic detection of blood by the Guajac method using Haemoccult, Beckman Coulter / PCD, Germany; 4: macroscopic blood visible), diarrhea (0: formed feces; 2: pasty feces; 4: liquid feces), and the clinical aspect (0: normal; 2: ruffled fur, less locomotion; 4: isolation, severely compromised locomotion, pre-final aspect) as described earlier (Heimesaat et al., 2014a).

### Sampling Procedures

At day 6 p.i., mice were sacrificed by isoflurane inhalation (Abbott, Germany). Luminal gastrointestinal samples (i.e., from stomach, duodenum, ileum and colon) and *ex vivo* biopsies from colon, MLN, liver, kidney, and spleen were taken under sterile conditions. Large intestinal samples were collected from each mouse in parallel for microbiological, immunohistopathological and immunological analyses. The absolute colonic lengths were measured by a ruler (in cm).

### Immunohistochemistry

*In situ* immunohistochemical analyses were performed in colonic *ex vivo* biopsies that had been immediately fixed in 5% formalin and embedded in paraffin as described earlier (Alutis et al., 2015a,b; Heimesaat et al., 2018). In brief, in order to detect apoptotic epithelial cells, proliferating epithelial cells, macrophages/monocytes, T lymphocytes and regulatory T cells, colonic paraffin sections (5 μm) were stained with primary antibodies directed against cleaved caspase 3 (Asp175, Cell Signaling, Beverly, MA, USA, 1:200), Ki67 (TEC3, Dako, Denmark, 1:100), F4/80 (# 14-4801, clone BM8, eBioscience, San Diego, CA, USA, 1:50), CD3 (#N1580, Dako, 1:10), and FOXP3 (FJK-16s, eBioscience, 1:100), respectively. Positively stained cells were then examined by light microscopy (magnification 100 x and 400 x), and for each mouse the average number of respective positively stained cells was determined within at least six high power fields (HPF, 0.287 mm^2^, 400 × magnification) by a blinded independent investigator.

### Bacterial Colonization

*C. jejuni* were quantitatively assessed in feces over time p.i. and in homogenates of *ex vivo* biopsies taken from MLN, spleen, liver, and kidney at day 6 p.i. by culture as stated elsewhere (Bereswill et al., 2011; Heimesaat et al., 2013). The detection limit of viable pathogens was ≈100 CFU per g.

### Cytokine Detection in Supernatants of Intestinal and Extra-Intestinal *ex vivo* Biopsies

Colonic *ex vivo* biopsies were cut longitudinally, washed in PBS, and strips of approximately 1 cm^2^ tissue were placed in 24-flat-bottom well culture plates (Nunc, Germany) containing 500 μL serum-free RPMI 1640 medium (Gibco, life technologies, UK) supplemented with penicillin (100 U/mL) and streptomycin (100 μg/mL; PAA Laboratories, Germany). After 18 h at 37°C, culture supernatants were tested for MCP-1, IL-6, TNF, and IFN-γ by the Mouse Inflammation Cytometric Bead Assay (CBA; BD Biosciences, Germany) on a BD FACSCanto II flow cytometer (BD Biosciences). Systemic pro-inflammatory cytokine concentrations were measured in serum samples.

### Statistical Analysis

Medians and levels of significance were determined using Mann-Whitney test (GraphPad Prism v7, USA) as indicated. Two-sided probability (p) values ≤ 0.05 were considered significant. Experiments were reproduced three times.

## Results

### Intestinal Colonization Properties of a *C. jejuni* 11168^HtrA-S197A^ Mutant Strain in Perorally Infected Secondary Abiotic IL-10^−/−^ Mice

**Figure 1:**
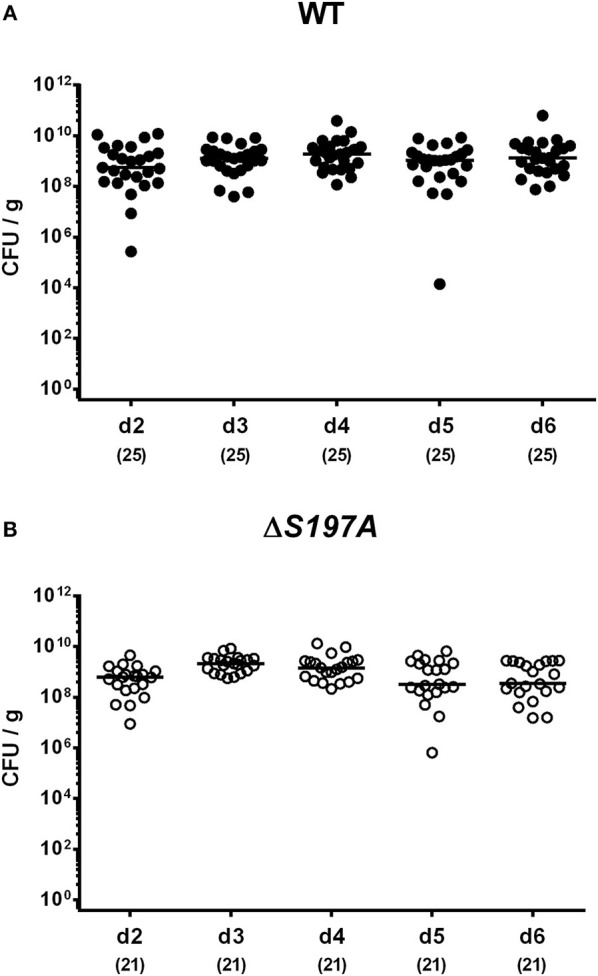
Time course of fecal *C. jejuni* loads following peroral infection of secondary abiotic IL-10^−/−^ mice. Secondary abiotic IL-10^−/−^ mice were perorally infected either with the *C. jejuni* 11168^WT^ strain [**(A)**, closed circles] or the isogenic *htrA* mutant 11168^HtrA−S197A^ [**(B)**, open circles] by gavage on two consecutive days (d) 0 and 1. Pathogenic loads were quantitated in fecal samples derived from each mouse on a daily basis post-infection by culture and expressed in colony forming units per g (CFU/g). Medians (black bars) and numbers of analyzed animals (in parentheses) are indicated. Data were pooled from four independent experiments.

**Figure 2:**
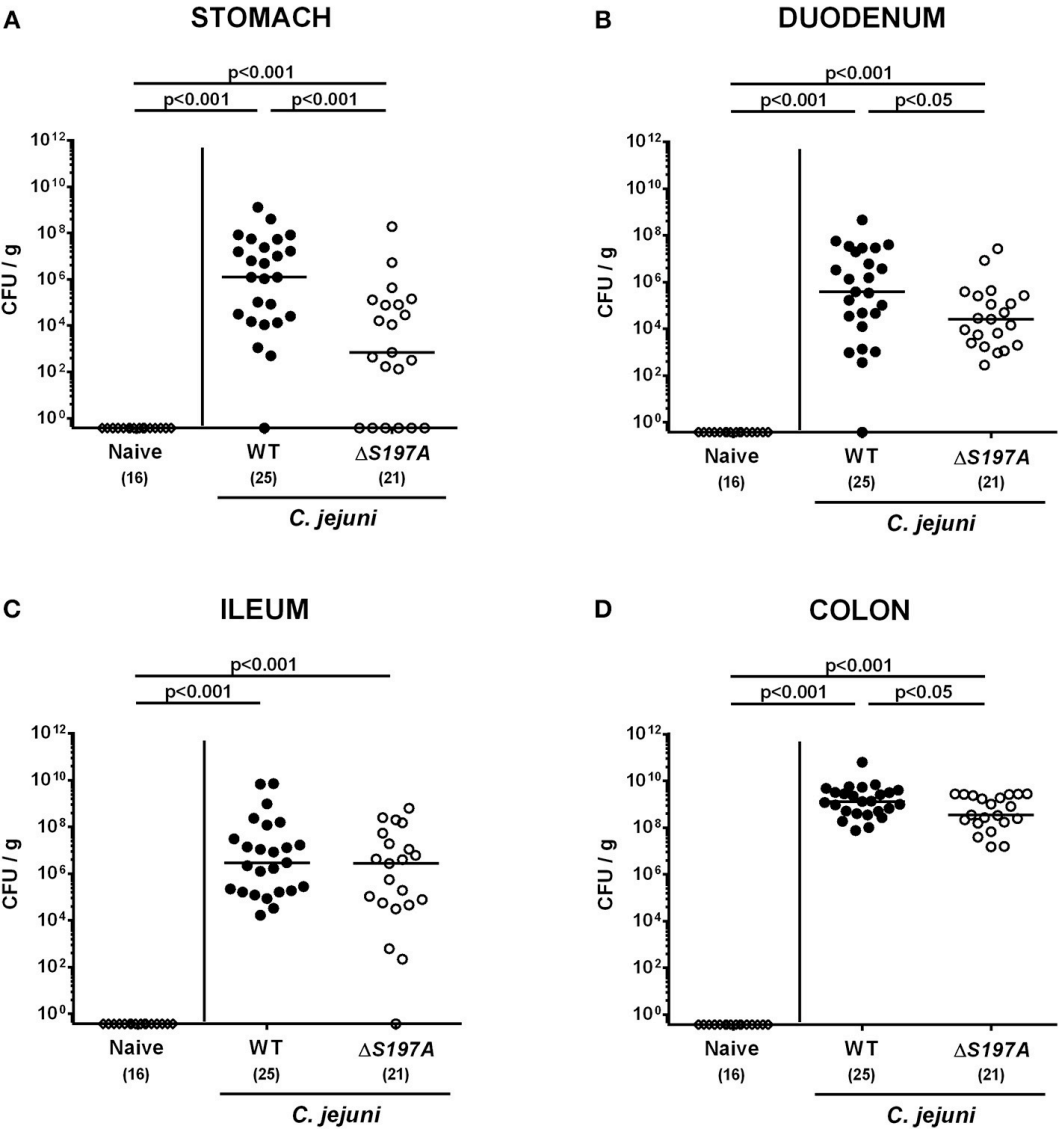
*C. jejuni* loads in the gastrointestinal tract of secondary abiotic IL-10^−/−^ mice six days following peroral infection. Secondary abiotic IL-10^−/−^ mice were perorally infected either with the *C. jejuni* 11168^WT^ strain (closed circles) or the isogenic *htrA* mutant 11168^HtrA−S197A^ (open circles) by gavage on days 0 and 1. Pathogenic loads (CFU, colony forming units per gram) were assessed in luminal samples derived from distinct parts of the gastrointestinal tract **(A–D)** at day 6 postinfection as indicated by culture. Naive mice (open diamonds) served as uninfected controls. Medians (black bars), levels of significance (*p*-values) determined by the Mann-Whitney *U*-test and numbers of analyzed animals (in parentheses) are indicated. Data were pooled from four independent experiments.

To assure stable *C. jejuni* infection, the gut microbiota and hence physiological colonization resistance of IL-10^−/−^ mice was abrogated by standard procedures using a ten-week course of broad-spectrum antibiotic treatment (Bereswill et al., 2011; Heimesaat et al., 2014a,b). Subsequently, generated secondary abiotic mice were subjected to peroral challenge with 10^9^ CFU of either the parental *C. jejuni* strain 11168^WT^ or its isogenic mutant 11168^HtrA−S197A^ variant. Our daily survey of fecal pathogen concentrations revealed that the point mutation in the *htrA* gene did not impact intestinal colonization properties as indicated by comparable median loads of 10^9^ CFU of either *C. jejuni* strain per g feces until day 6 p.i. (Figure 1). Upon sacrifice, the 11168^HtrA−S197A^ mutant strain infected mice harbored lower *C. jejuni* numbers in luminal samples taken from the stomach, duodenum and colon as compared to 11168^WT^ infected counterparts (*p* < 0.05–0.001; Figure 2). Of note, the differences in *C. jejuni* loads were less pronounced the more distal the gastrointestinal compartment the samples were derived from. Particularly in the colonic lumen the differences in median pathogen loads were approximately 0.5 orders of magnitude and hence rather minor (*p* < 0.05; Figure 2D).

### Macroscopic Sequelae in *C. jejuni* 11168^HtrA-S197A^ Infected Secondary Abiotic IL-10^−/−^ Mice

**Figure 3:**
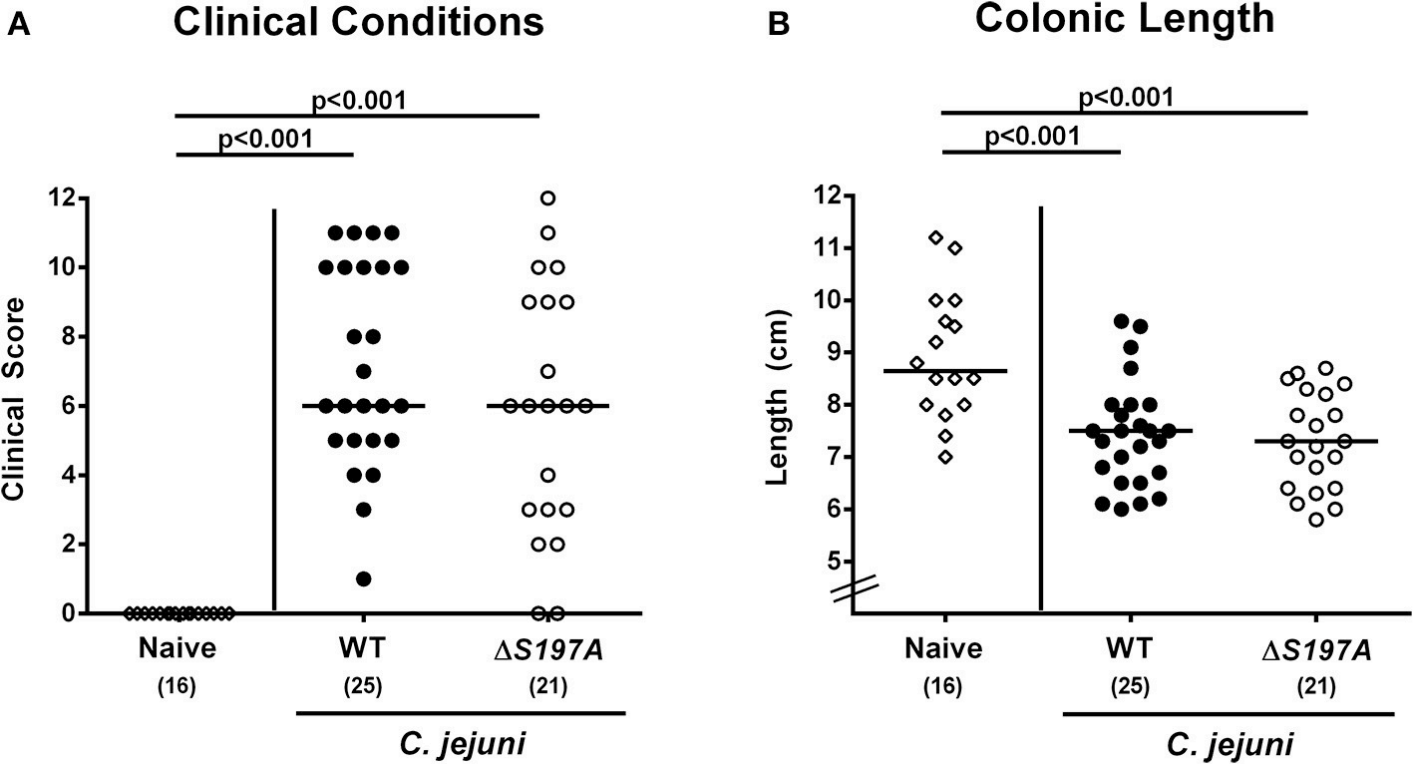
Macroscopic sequelae in *C. jejuni* infected secondary abiotic IL-10^−/−^ mice. Secondary abiotic IL-10^−/−^ mice were perorally infected either with the *C. jejuni* 11168^WT^ strain (closed circles) or the isogenic *htrA* mutant 11168^HtrA−S197A^ (open circles) by gavage on days 0 and 1. **(A)** Clinical symptoms were quantitatively assessed applying a standardized clinical scoring system on day 6 postinfection (see methods) and **(B)** colonic lengths were measured by a ruler (in cm). Naive mice (open diamonds) served as uninfected controls. Medians (black bars), level of significance (*p*-values) as determined by Mann-Whitney *U*-test and numbers of analyzed animals (in parentheses) are indicated. Data were pooled from four independent experiments.

We further assessed whether the disease development upon peroral infection of secondary abiotic IL-10^−/−^ mice differed between 11168^HtrA−S197A^ mutant and 11168^WT^ strains. Until day 6 following infection with either strain, mice were suffering from comparably severe *C. jejuni* induced disease as indicated by similar clinical scores that had been assessed during our kinetic survey (Figure 3A and Figure S1). We further stratified clinical conditions of *C. jejuni* infected mice to fecal blood positivity rates and determined virtually comparably frequent abundances of fecal blood following infection with either *C. jejuni* strain (Figure S2). Given that intestinal inflammation is accompanied by significant shortening of the inflamed intestine (Heimesaat et al., 2006, 2007; Haag et al., 2012a,b), we measured the large intestinal lengths upon necropsy. At day 6 p.i., colonic lengths of mice infected with either strain were comparable (n.s. Figure 3B), but lower as compared to uninfected naive control animals as expected (*p* < 0.001; Figure 3B). Hence, the loss of proteolytic activity of HtrA caused by the S197A point mutation in the *htrA* gene did neither impact pathogenic intestinal colonization properties nor gross disease following peroral *C. jejuni* infection of secondary abiotic IL-10^−/−^ mice.

### Intestinal Histopathology and Inflammatory Responses in *C. jejuni* 11168^HtrA-S197A^ Infected Secondary Abiotic IL-10^−/−^ Mice

**Figure 4:**
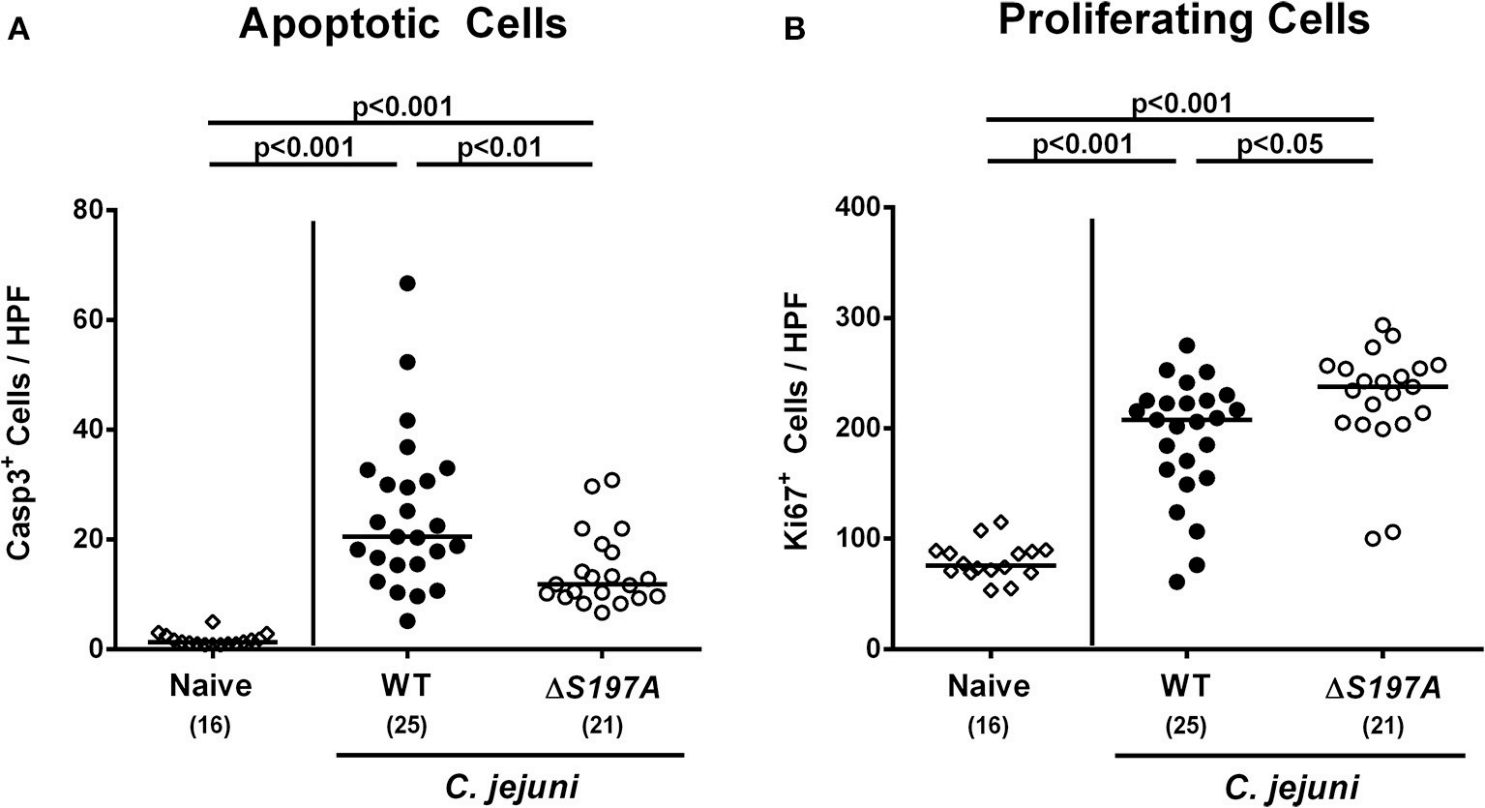
Microscopic colonic sequelae in *C. jejuni* infected secondary abiotic IL-10^−/−^ mice. Secondary abiotic IL-10^−/−^ mice were perorally infected either with the *C. jejuni* 11168^WT^ strain (closed circles) or the isogenic *htrA* mutant 11168^HtrA−S197A^ (open circles) by gavage on days 0 and 1. The average numbers of colonic epithelial **(A)** apoptotic cells (positive for caspase-3, Casp3) and **(B)** proliferating/regenerating cells (positive for Ki67) from six high power fields (HPF, 400x magnification) per animal were assessed microscopically in immunohistochemically stained large intestinal paraffin sections at day 6 postinfection. Naive mice (open diamonds) served as uninfected controls. Medians (black bars), levels of significance (*p*-values) determined by the Mann-Whitney *U*-test and numbers of analyzed animals (in parentheses) are indicated. Data were pooled from four independent experiments.

We next surveyed inflammatory sequelae of *C. jejuni* infection histologically. At day 6 p.i., mice infected with either strain exhibited multi-fold increased numbers of apoptotic epithelial cells as compared to uninfected counterparts (*p* < 0.001 vs. naive; Figure 4A and Figure S3A). This increase was, however, less pronounced in mice infected with the 11168^HtrA−S197A^ mutant as compared to the 11168^WT^ infected animals (*p* < 0.01; Figure 4A and Figure S3A), given that the former displayed approximately 50% of caspase3 positive colonic epithelial cells as compared to the latter (Figure 4A and Figure S3A). Furthermore, *C. jejuni* infection resulted in increased numbers of Ki67 positive colonic epithelial cells indicative for enhanced cell proliferation and regeneration (p < 0.001 vs. naive; Figure 4B and Figure S3B). Conversely to the caspase3 positive cells, 11168^HtrA−S197A^ infected mice displayed slightly higher Ki67 positive cells in the large intestinal epithelium as compared to 11168^WT^ strain infected counterparts at day 6 p.i. (Figure 4B and Figure S3B).

**Figure 5:**
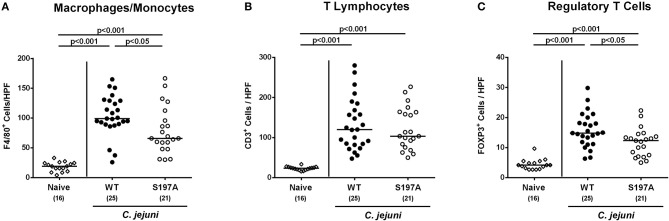
Colonic immune cell responses in *C. jejuni* infected secondary abiotic IL-10^−/−^ mice. Secondary abiotic IL-10^−/−^ mice were perorally infected either with the *C. jejuni* 11168^HtrA−S197A^ strain (closed circles) or the isogenic *htrA* mutant 11168^HtrA−S197A^ (open circles) by gavage on days 0 and 1. At day 6 postinfection, the average numbers of colonic epithelial of colonic **(A)** macrophages and monocytes (positive for F4/80), **(B)** T lymphocytes (positive for CD3), and **(C)** regulatory T cells (positive for FOXP3) were assessed microscopically from six high power fields (HPF, 400x magnification) per animal in immunohistochemically stained large intestinal paraffin sections. Naive mice (open diamonds) served as uninfected controls. Medians (black bars), levels of significance (*p*-values) determined by the Mann-Whitney *U*-test and numbers of analyzed animals (in parentheses) are indicated. Data were pooled from four independent experiments.

We further assessed innate (such as F4/80 positive macrophages and monocytes) and adaptive (including CD3 positive T lymphocytes and FOXP3 positive regulatory T cells) immune responses upon *C. jejuni* infection. At day 6 p.i., increased cell numbers of both adaptive and innate immunity parameters could be detected in the mucosa and lamina propria of infected mice irrespective of the applied *C. jejuni* strain (Figure 5 and Figure S3). In the colon of 11168^HtrA−S197A^ infected mice, however, lower numbers of F4/80 positive and FOXP3 positive cells could be determined as compared to 11168^WT^ infected control mice (*p* < 0.05; Figures 5A,C and Figures S3C,E). Hence, the protease-inactive *htrA* point mutant *C. jejuni* strain caused less pronounced colonic apoptosis and immune cell responses, but enhanced epithelial proliferation upon peroral infection of secondary abiotic IL-10^−/−^ mice.

### Colonic and Systemic Pro-Inflammatory Cytokine Production in *C. jejuni* 11168^HtrA-S197A^ Infected Secondary Abiotic IL-10^−/−^ Mice

**Figure 6:**
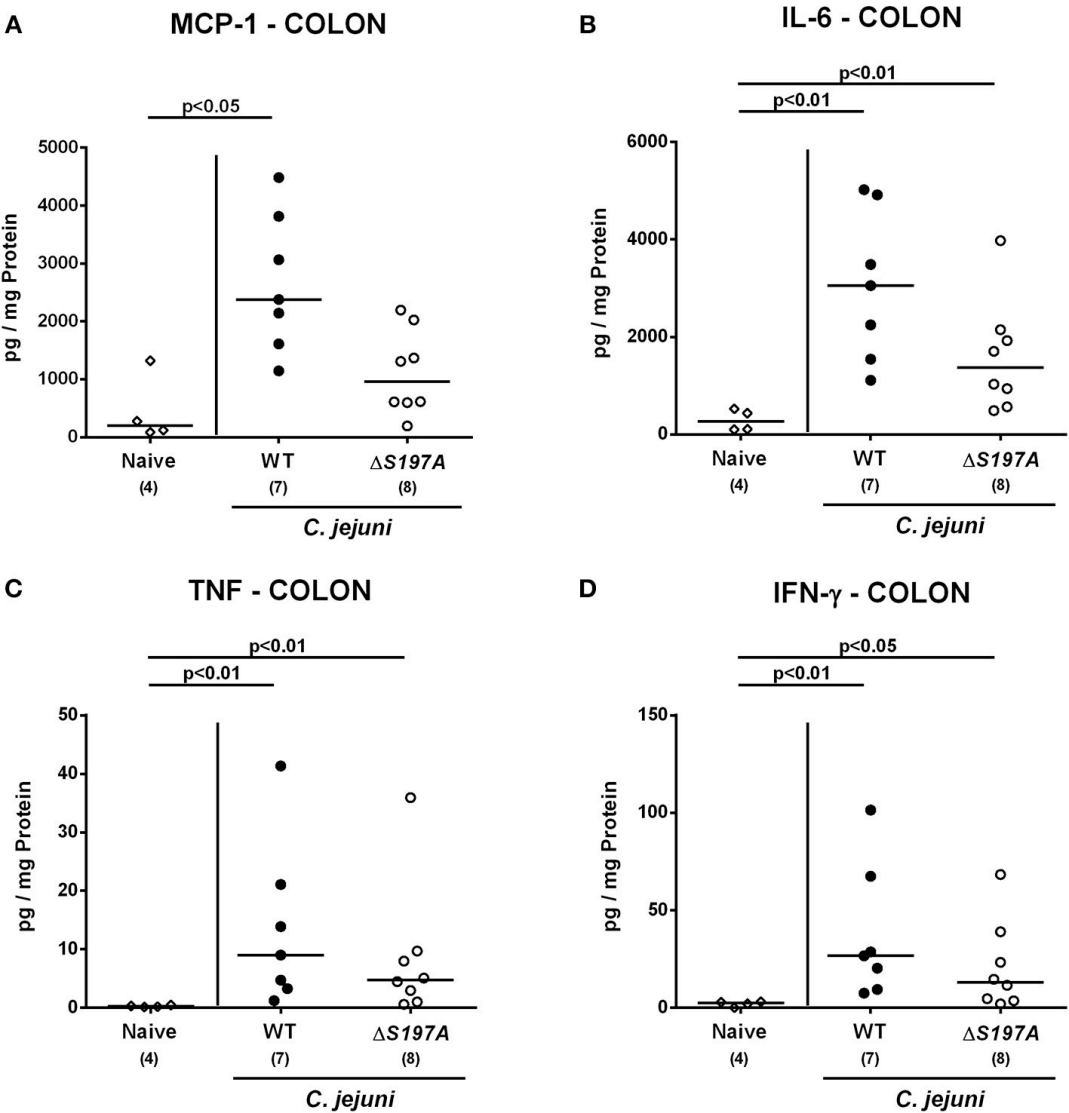
Colonic secretion of pro-inflammatory cytokines in *C. jejuni* infected secondary abiotic IL-10^−/−^ mice. Secondary abiotic IL-10^−/−^ mice were perorally infected either with the *C. jejuni* 11168^HtrA−S197A^ strain (closed circles) or the isogenic *htrA* mutant 11168^HtrA−S197A^ (open circles) by gavage on days 0 and 1. **(A)** MCP-1, **(B)** IL-6, **(C)** TNF, and **(D)** IFN-γ concentrations were measured in supernatants derived from colonic *ex vivo* biopsies at day 6 postinfection. Naive mice (open diamonds) served as uninfected controls. Medians (black bars), levels of significance (*p*-value) determined by the Mann-Whitney *U*-test and numbers of analyzed animals (in parentheses) are indicated. Data representative for four independent experiments are shown.

**Figure 7:**
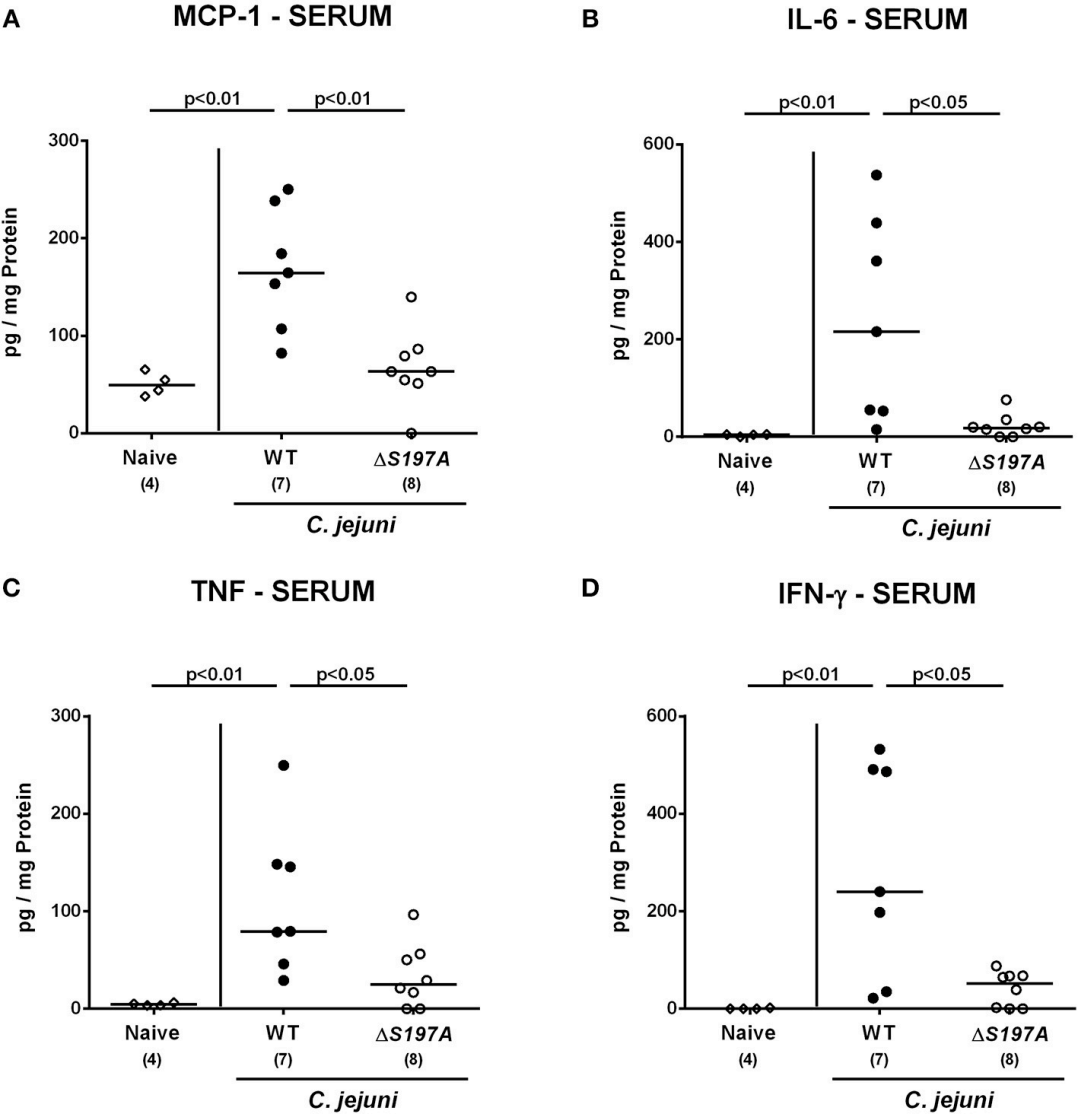
Systemic secretion of pro-inflammatory cytokines in *C. jejuni* infected secondary abiotic IL-10^−/−^ mice. Secondary abiotic IL-10^−/−^ mice were perorally infected either with the *C. jejuni* 11168^HtrA−S197A^ strain (closed circles) or the isogenic *htrA* mutant 11168^HtrA−S197A^ (open circles) by gavage on days 0 and 1. **(A)** MCP-1, **(B)** IL-6, **(C)** TNF, and **(D)** IFN-γ concentrations were measured in serum samples taken at day 6 postinfection. Naive mice (open diamonds) served as uninfected controls. Medians (black bars), levels of significance (*p*-value) determined by the Mann-Whitney *U*-test and numbers of analyzed animals (in parentheses) are indicated. Data representative for four independent experiments are shown.

We next surveyed pro-inflammatory cytokine concentrations upon infection with respective *C. jejuni* strains in colonic *ex vivo* biopsies (Figure 6). Six days following peroral challenge, large intestinal MCP-1 concentrations induced by the 11168^HtrA−S197A^ strain were lower as compared to those in mice infected with *C. jejuni* 11168^WT^(*p* < 0.05 vs. naive; Figure 6A). In addition, *C. jejuni* infection with either strain resulted in elevated colonic IL-6, TNF and IFN-γ secretion, whereas a trend toward lower IL-6 concentrations could be assessed in the large intestines of 11168^HtrA−S197A^ as compared to 11168^WT^ strain infected mice at day 6 p.i. (n.s.; Figure 6B). Strikingly, strain-dependent pro-inflammatory cytokine secretion was not restricted to the gut, given that pro-inflammatory cytokines such as MCP-1, IL-6, TNF, and IFN-γ in serum samples taken at day 6 p.i. were lower in mice infected with the 11168^HtrA−S197A^ strain as compared to mice challenged with *C. jejuni* 11168^WT^ (*p* < 0.05–0.01; Figure 7).

We further addressed whether the lowered immune responses observed in mice challenged with the 11168^HtrA−S197A^ mutant had a consequence for transepithelial migration and subsequent spread of viable *C. jejuni* from the intestinal tract to extra-intestinal including systemic compartments. Notably, at day 6 p.i., *C. jejuni* could be cultured from 40% and 75% of MLN taken from 11168^WT^ and 11168^HtrA−S197A^ strain infected mice, respectively (Figure S4). Furthermore, viable *C. jejuni* could be isolated from extra-intestinal and systemic compartments such as liver, kidney, spleen and cardiac blood in single cases only (Figure S4). Interestingly, in 14.3% of 11168^HtrA−S197A^ strain infected mice, but none of 11168^WT^ samples, viable pathogens were detectable in cardiac blood (Figure S4). Hence, the *C. jejuni* 11168^HtrA−S197A^ strain translocated with higher efficiency possibly as a result of less distinct intestinal, but also less pronounced systemic pro-inflammatory cytokine secretion as compared to the 11168^WT^ strain.

Taken together, investigation of the 11168^HtrA−S197A^ mutant strain in the secondary abiotic IL-10^−/−^ mice infection model revealed that the protease activity of *C. jejuni* HtrA is not required for intestinal colonization and establishment of disease, but triggers apoptosis and pro-inflammatory immune responses in the intestines as well as in systemic compartments. It is of note that the proteolytic activity of HtrA limits bacterial translocation to MLN and thus protects the host against systemic spread of *C. jejuni*.

## Discussion

The serine protease and chaperone HtrA constitutes a remarkable virulence factor that enables *C. jejuni* to transmigrate across polarized epithelial cells by cleavage of E-cadherin and probably other junctional proteins (Boehm et al., 2012, 2018; Hoy et al., 2012). HtrA chaperone activity seems to be crucial for *C. jejuni* adherence to epithelial host cells and subsequently invasion, whereby HtrA protease activity appears to be not essential for these processes (Bæk et al., 2011). We have recently shown in infected mice, that the serine protease HtrA is not required for induction of enterocolitis by *C. jejuni per se* and plays a role in the modulation of apoptosis and immunopathology during campylobacteriosis (Heimesaat et al., 2014a,b). Six days after infection with the *C. jejuni* Δ*htrA* deletion mutant, secondary abiotic IL-10^−/−^ mice were suffering from less severe *C. jejuni* induced symptoms and developed less pronounced intestinal as well as extra-intestinal including systemic pro-inflammatory immune responses upon infection as compared to 11168^WT^ strain infected control mice suffering from acute enterocolitis (Heimesaat et al., 2014a,b). In order to unravel in particular the role of the protease activity of HtrA in colonization efficiency, clinical symptoms as well as intestinal and extra-intestinal inflammation in more detail, we analyzed the *C. jejuni* 11168^HtrA−S197A^ point mutant variant in secondary abiotic IL-10^−/−^ mice in the present study. This mutant lacks protease activity, but still retains the chaprone function (Bæk et al., 2011; Boehm et al., 2012). Six days following peroral infection, mice harbored comparable pathogen loads of the mutant strain 11168^HtrA−S197A^ or the parental 11168^WT^ strain. Hence, inactivation of the serine protease active site did not alter colonic colonization capacity of *C. jejuni*. This supports the findings of Bæk and co-workers that the periplasmic chaperone activity of HtrA is more important for efficient binding to epithelial host cells than its protease activity (Bæk et al., 2011). More importantly, *in vitro* studies revealed that the protease-deficient S197A point mutant is incapable of transmigrating across polarized epithelial cells (Boehm et al., 2012). These findings led to the hypothesis, that the mutant expressing the 11168^HtrA−S197A^ variant would rather not induce intestinal immunopathology of campylobacteriosis. However, our present *in vivo* study revealed that secondary abiotic IL-10^−/−^ mice infected with the 11168^HtrA−S197A^ strain did in fact develop ulcerative enterocolitis, which was characterized by bloody diarrhea and wasting symptoms and were comparable to the symptoms observed following 11168^WT^ strain infection. This observation is surprising and suggests that in addition to HtrA other yet unknown bacterial factors contribute to transepithelial migration of *C. jejuni* and disease development in mice.

Interestingly, mice infected with the *C. jejuni* 11168^HtrA−S197A^ strain displayed less immunopathological sequelae within the intestinal tract including colonic epithelial apoptosis when compared to the 11168^WT^ strain infected mice, which is in contrast to the macroscopic phenotype that was similar in 11168^WT^ strain infection. One needs to take into consideration, however, that the macroscopic aspect (i.e., the clinical symptoms) of the mice are rather the sum of many different pathophysiological sequelae from *C. jejuni* infection and cannot only be attributed to the immune responses and immunopathological features observed microscopically in the intestinal mucosa. In fact, cell proliferative and hence regenerative measures observed in colonic epithelia, thus counteracting *C. jejuni* induced cell damage including colonic epithelial apoptosis, were more pronounced in *C. jejuni* 11168^HtrA−S197A^ strain as compared to parental strain infected mice. Thus, protease activity of HtrA triggers colonic apoptosis, but dampens cell proliferation required for tissue regeneration. Similar results were obtained in our previous analysis of the *htrA* deletion mutant Δ*htrA* (Heimesaat et al., 2014a,b), which further supports that the protease activity of HtrA rather inhibits regeneration of damaged tissue and thereby further aggravates intestinal inflammation. This let us to propose that secreted HtrA might target immunoregulatory factor(s) directly, and future experiments should address this hypothesis. The idea that the protease activity of HtrA triggers inflammatory processes in host cells is further underlined by lower numbers of immune cells at the site of infection and less secretion of pro-inflammatory cytokines in intestinal, but also extra-intestinal and, remarkably, even systemic compartments following *C. jejuni* 11168^HtrA−S197A^ as compared to 11168^WT^ strain infection as shown here and in our previous studies with the *C. jejuni* Δ*htrA* mutant vs. parental strain (Heimesaat et al., 2014a,b). Of note, immune responses triggered by the protease activity of HtrA prevented translocation of viable pathogens from the intestinal lumen to MLN and possibly to extra-intestinal including systemic compartments. However, on the systemic level live bacteria could be isolated, if at all, in single case only, irrespective of the applied strain.

Taken together, our data demonstrate that the proteolytic activity of HtrA results in the amplification of host immune responses upon *C. jejuni* infection of mice, but is not required for the onset and establishment of intestinal disease. Future studies will unravel if further distinct domains within the HtrA protein might have functional immunopathological implications during campylobacteriosis.

## Data Availability

All datasets generated for this study are included in the manuscript and/or the Supplementary Files.

## Ethics Statement

All animal experiments were conducted in accordance with the European Guidelines for animal welfare (2010/63/EU) following approval of the protocol by the commission for animal experiments headed by the “Landesamt für Gesundheit und Soziales” (LaGeSo, Berlin, registration number G0172/16). Clinical conditions of mice were assessed twice a day.

## Author Contributions

A-MS: designed and performed experiments, analyzed data, co-wrote paper; SM: performed experiments, analyzed data; UE: performed experiments; MB: provided bacterial strains, co-edited paper; SBa: provided advice in experimental design, critically discussed results, co-edited paper; SBe: provided advice in experimental design, critically discussed results, co-edited paper; MH: designed and performed experiments, analyzed data, wrote paper.

### Conflict of Interest Statement

The authors declare that the research was conducted in the absence of any commercial or financial relationships that could be construed as a potential conflict of interest.

## Supplementary Material

Figure S1 Kinetic survey of clinical conditions in *C. jejuni* infected secondary abiotic IL-10^−/−^ mice. Secondary abiotic IL-10^−/−^ mice were perorally infected either with the *C. jejuni* 11168^WT^ strain (**A**, closed circles) or the isogenic *htrA* mutant 11168^HtrA−S197A^ (**B**, open circles) by gavage on day (d) 0 and d1. Clinical symptoms were quantitatively assessed applying a standardized clinical scoring system from d0 until d6 postinfection (see methods). Medians (black bars) and numbers of analyzed mice (in parentheses) are indicated. Data were pooled from four independent experiments.

Figure S2 Kinetic survey of fecal blood in *C. jejuni* infected secondary abiotic IL-10^−/−^ mice. Secondary abiotic IL-10^−/−^ mice were perorally infected either with the *C. jejuni* 11168^WT^ strain (**A**, black bars) or the isogenic *htrA* mutant 11168^HtrA−S197A^ (**B**, white bars) by gavage on days (d) 0 and 1. The cumulative relative abundance of fecal blood (fecal blood^+^) out of four independent experiments was assessed macroscopically and microscopically (in %). The numbers of fecal blood positive mice out of the total numbers of analyzed animals are given in parentheses at defined time points.

Figure S3 Representative photomicrographs illustrating apoptotic and proliferating epithelial as well as immune cells responses in large intestines of *C. jejuni* infected secondary abiotic IL-10^−/−^ mice. Secondary abiotic IL-10^−/−^ mice were perorally infected either with the strain *C. jejuni* 11168^WT^ or the isogenic *htrA* mutant 11168^HtrA−S197A^ by gavage on days 0 and 1. Naive mice served as uninfected controls. Photomicrographs representative for four independent experiments illustrate the average numbers of **(A)** apoptotic epithelial cells (Casp3+), **(B)** proliferating epithelial cells, **(C)** macrophages and monocytes (F4/80+), **(D)** T lymphocytes (CD3+), and **(E)** regulatory T cells (FOXP3+) in at least six high power fields (HPF) as quantitatively assessed in colonic paraffin sections applying *in situ* immunohistochemistry at day 6 postinfection (100 × magnification, scale bar 100 μm).

Figure S4 Bacterial translocation in *C. jejuni* infected secondary abiotic IL-10^−/−^ mice. Secondary abiotic IL-10^−/−^ mice were perorally infected either with the *C. jejuni* strain 11168^WT^ (black bars) or the isogenic *htrA* mutant 11168^HtrA−S197A^ (open bars) by gavage on days 0 and 1. At day 6 postinfection, pathogenic loads were quantitatively assessed in *ex vivo* biopsies derived from **(A)** MLN, **(B)** liver, **(C)** kidney, **(D)** spleen and **(E)** cardiac blood by culture. The cumulative relative translocation rate of viable pathogens into the respective compartment out of four independent experiments is indicated (in %).

## Abbreviations

CBA: Cytometric Bead Array
CFU: colony-forming units
HtrA: high-temperature requirement A
i.e.: *id est*
IFN: interferon
IL: interleukin
MAPK/ERK: mitogen-activated protein kinases/extracellular signal-regulated kinases
MCP: Monocyte chemoattractant protein
MLN: mesenteric lymph nodes
n.s.: not significant
NF-κB: nuclear factor kappa-light-chain-enhancer of activated B cells
Nod: nucleotide-oligomerization-domain
PBS: phosphate-buffered saline
PDZ, post synaptic density protein (PSD59), Drosophila disc large tumor suppressor (Dlg1): zonula occludens-1 protein (ZO-1)
p: probability
p.i.: postinfection
SPF: specific pathogen free
TLR: toll-like receptor
TNF: tumor necrosis factor
WT: wildtype.
