# Supplementary figures and images for: Protease Activity of Campylobacter jejuni HtrA Modulates Distinct Intestinal and Systemic Immune Responses in Infected Secondary Abiotic IL-10 Deficient Mice
Source: Front Cell Infect Microbiol. 2019 Mar 29;9:79. doi: 10.3389/fcimb.2019.00079 (PMC6449876; doi:10.3389/fcimb.2019.00079)

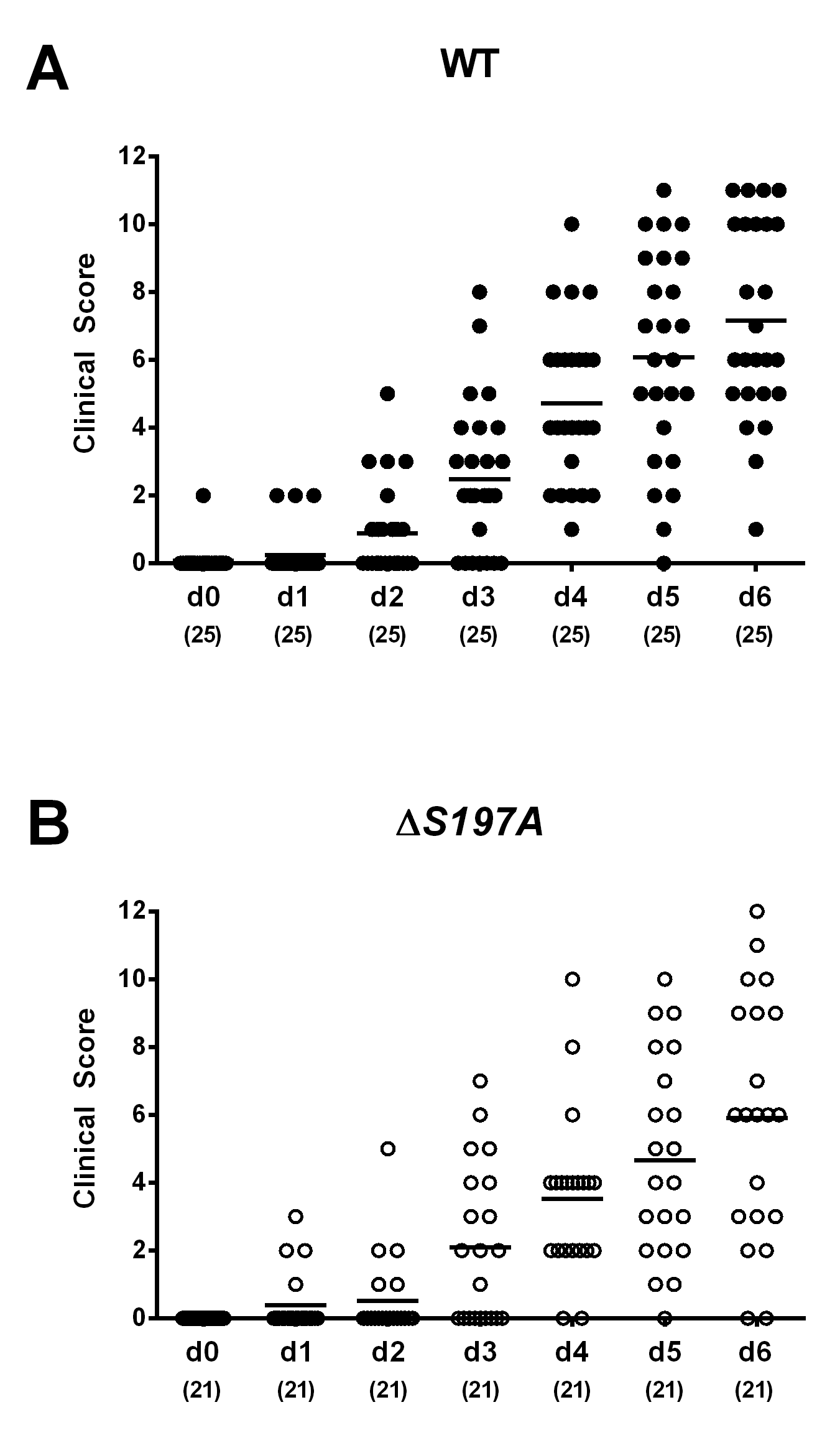

Supplement: Figure S1 — Kinetic survey of clinical conditions in C. jejuni infected secondary abiotic IL-10−/− mice. Secondary abiotic IL-10−/− mice were perorally infected either with the C. jejuni 11168WT strain (A, closed circles) or the isogenic htrA mutant 11168HtrA−S197A (B, open circles) by gavage on day (d) 0 and d1. Clinical symptoms were quantitatively assessed applying a standardized clinical scoring system from d0 until d6 postinfection (see methods). Medians (black bars) and numbers of analyzed mice (in parentheses) are indicated. Data were pooled from four independent experiments. [file Image_1.tiff]

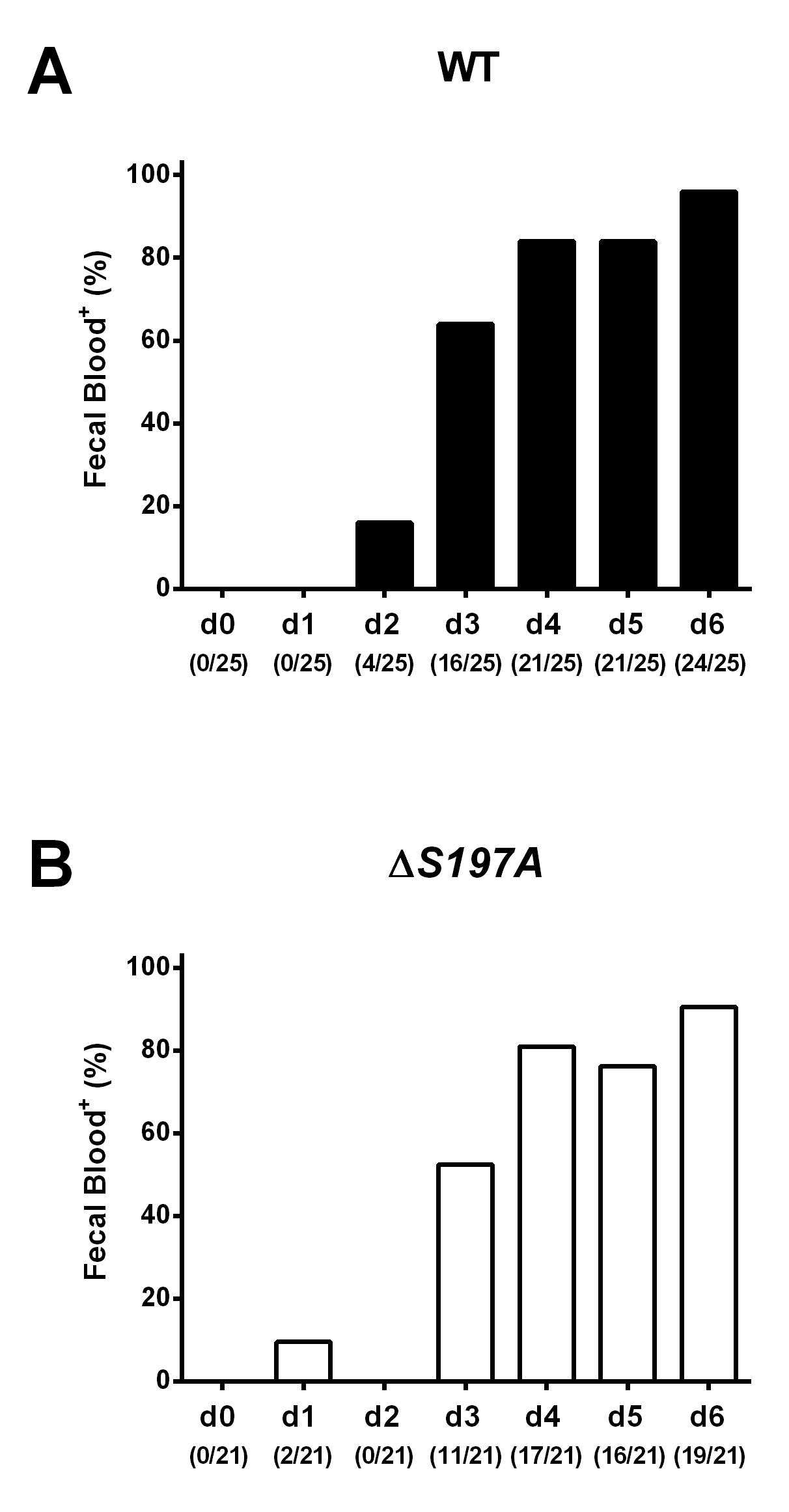

Supplement: Figure S2 — Kinetic survey of fecal blood in C. jejuni infected secondary abiotic IL-10−/− mice. Secondary abiotic IL-10−/− mice were perorally infected either with the C. jejuni 11168WT strain (A, black bars) or the isogenic htrA mutant 11168HtrA−S197A (B, white bars) by gavage on days (d) 0 and 1. The cumulative relative abundance of fecal blood (fecal blood+) out of four independent experiments was assessed macroscopically and microscopically (in %). The numbers of fecal blood positive mice out of the total numbers of analyzed animals are given in parentheses at defined time points. [file Image_2.tiff]

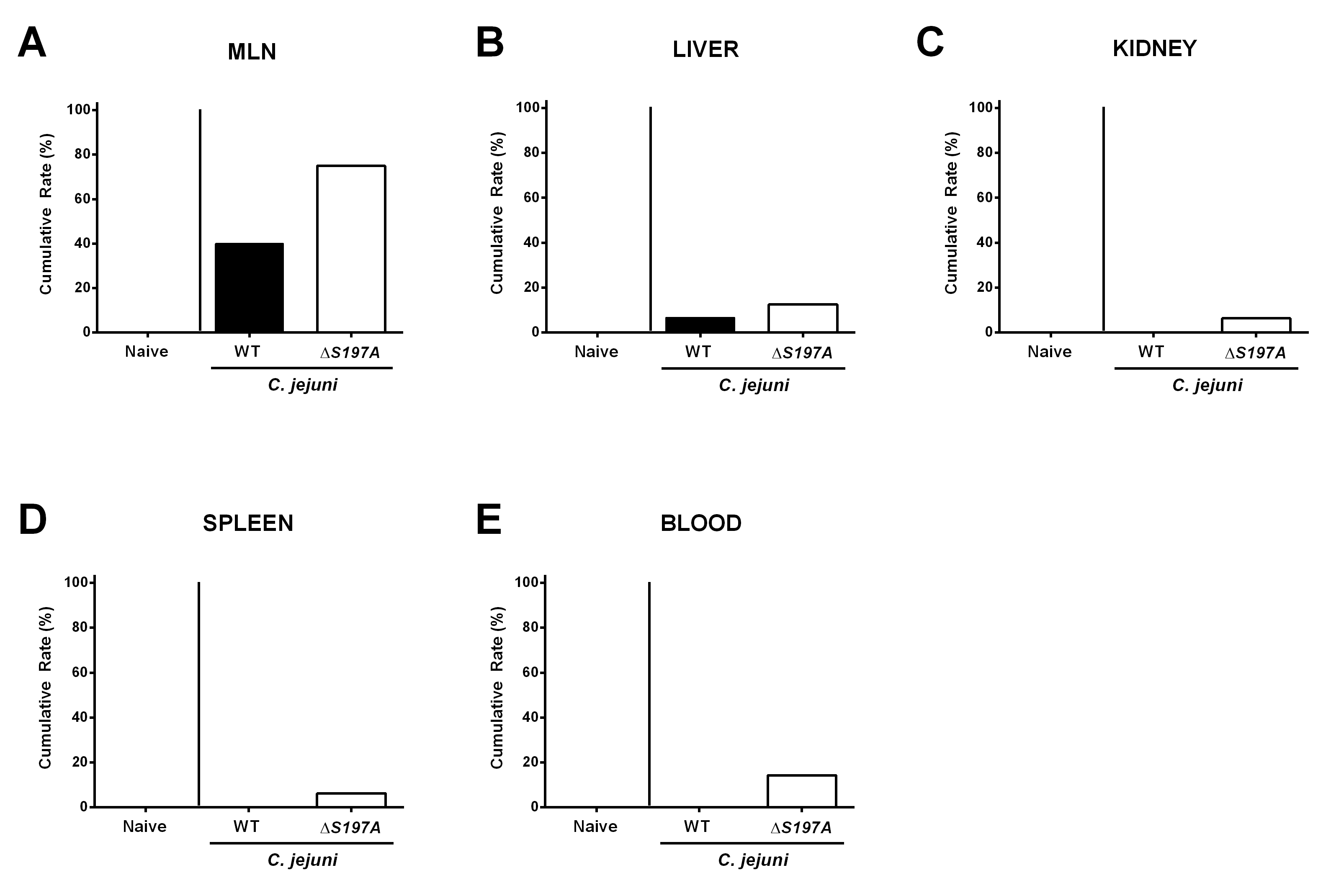

Supplement: Figure S4 — Bacterial translocation in C. jejuni infected secondary abiotic IL-10−/− mice. Secondary abiotic IL-10−/− mice were perorally infected either with the C. jejuni strain 11168WT (black bars) or the isogenic htrA mutant 11168HtrA−S197A (open bars) by gavage on days 0 and 1. At day 6 postinfection, pathogenic loads were quantitatively assessed in ex vivo biopsies derived from (A) MLN, (B) liver, (C) kidney, (D) spleen and (E) cardiac blood by culture. The cumulative relative translocation rate of viable pathogens into the respective compartment out of four independent experiments is indicated (in %). [file Image_4.tiff]
