# Supplementary material for: Protease Activity of Campylobacter jejuni HtrA Modulates Distinct Intestinal and Systemic Immune Responses in Infected Secondary Abiotic IL-10 Deficient Mice
Source: Front Cell Infect Microbiol. 2019 Mar 29;9:79. doi: 10.3389/fcimb.2019.00079 (PMC6449876; doi:10.3389/fcimb.2019.00079)
Supplement: Figure S3 — Representative photomicrographs illustrating apoptotic and proliferating epithelial as well as immune cells responses in large intestines of C. jejuni infected secondary abiotic IL-10−/− mice. Secondary abiotic IL-10−/− mice were perorally infected either with the strain C. jejuni 11168WT or the isogenic htrA mutant 11168HtrA−S197A by gavage on days 0 and 1. Naive mice served as uninfected controls. Photomicrographs representative for four independent experiments illustrate the average numbers of (A) apoptotic epithelial cells (Casp3+), (B) proliferating epithelial cells, (C) macrophages and monocytes (F4/80+), (D) T lymphocytes (CD3+), and (E) regulatory T cells (FOXP3+) in at least six high power fields (HPF) as quantitatively assessed in colonic paraffin sections applying in situ immunohistochemistry at day 6 postinfection (100 × magnification, scale bar 100 μm). [file Image_3.pdf]

# A Apoptotic Cells (COLON)

**Naive** | ***C. jejuni* WT** | ***C. jejuni* ΔS197A**

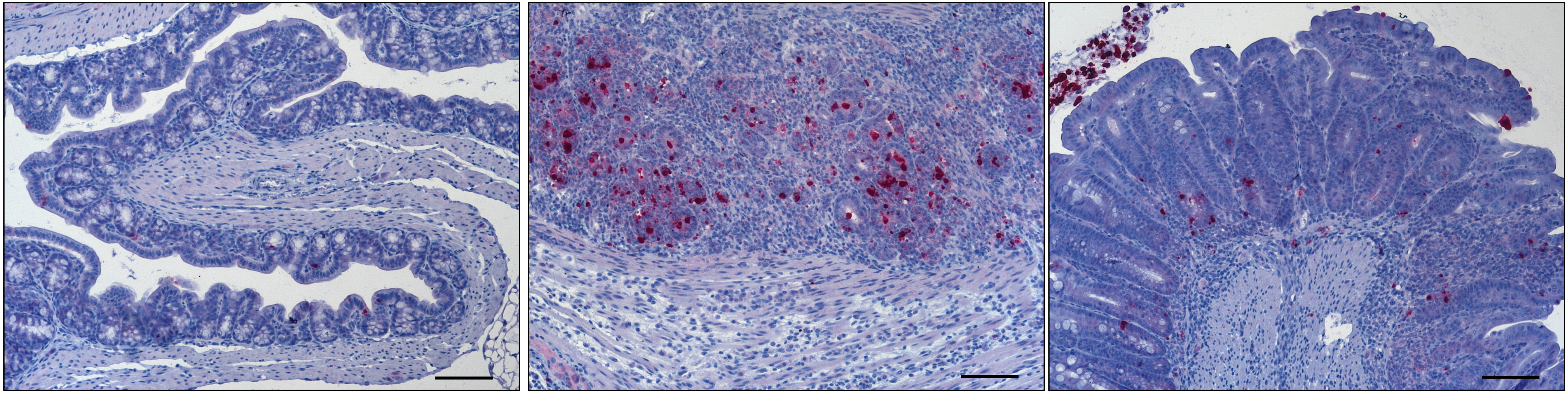

**(100 x magnification, scale bar 100 µm)**

**B** Proliferating Cells (COLON)

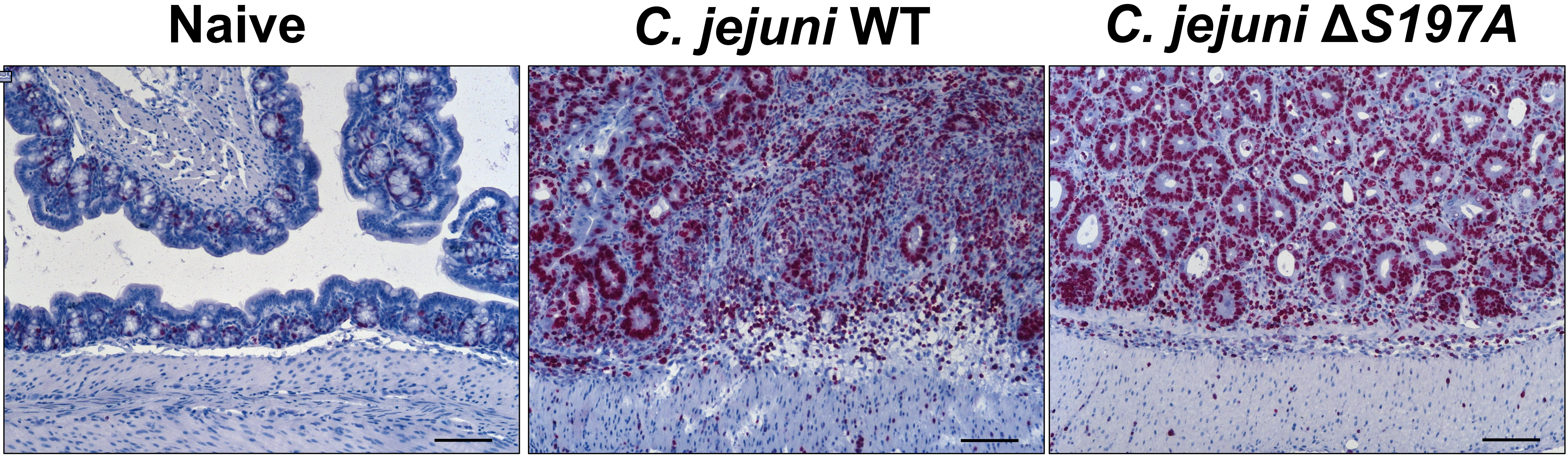


(100 x magnification, scale bar 100 µm)

# C Macrophages / Monocytes (COLON)

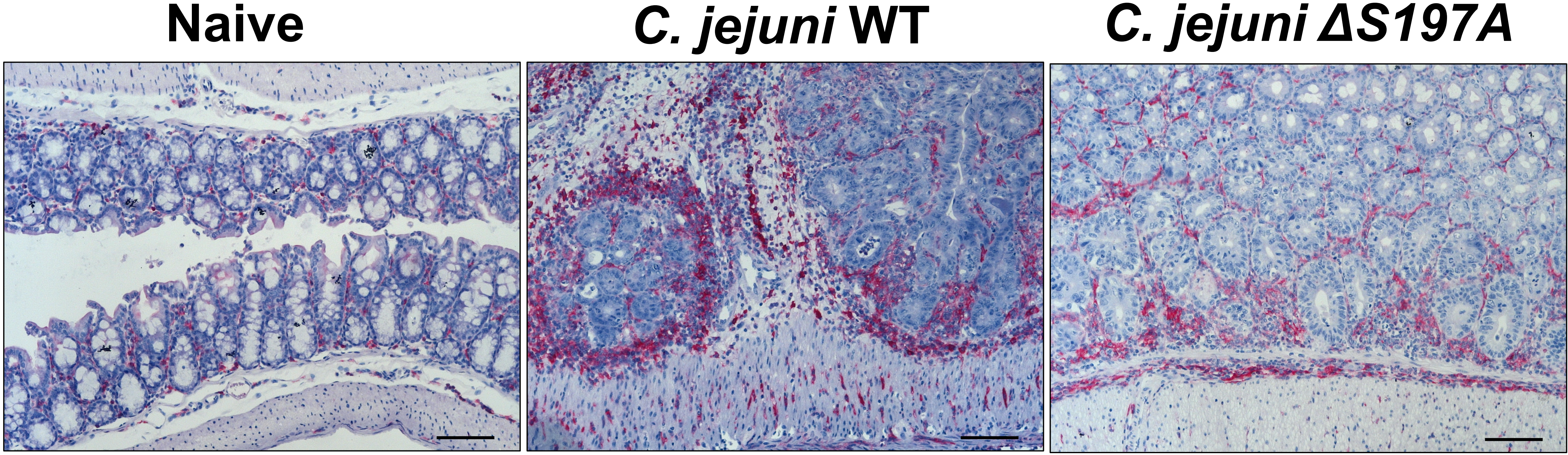


(100 x magnification, scale bar 100 µm)

**D** **T Lymphocytes (COLON)**

**Naive** | ***C. jejuni* WT** | ***C. jejuni ΔS197A***

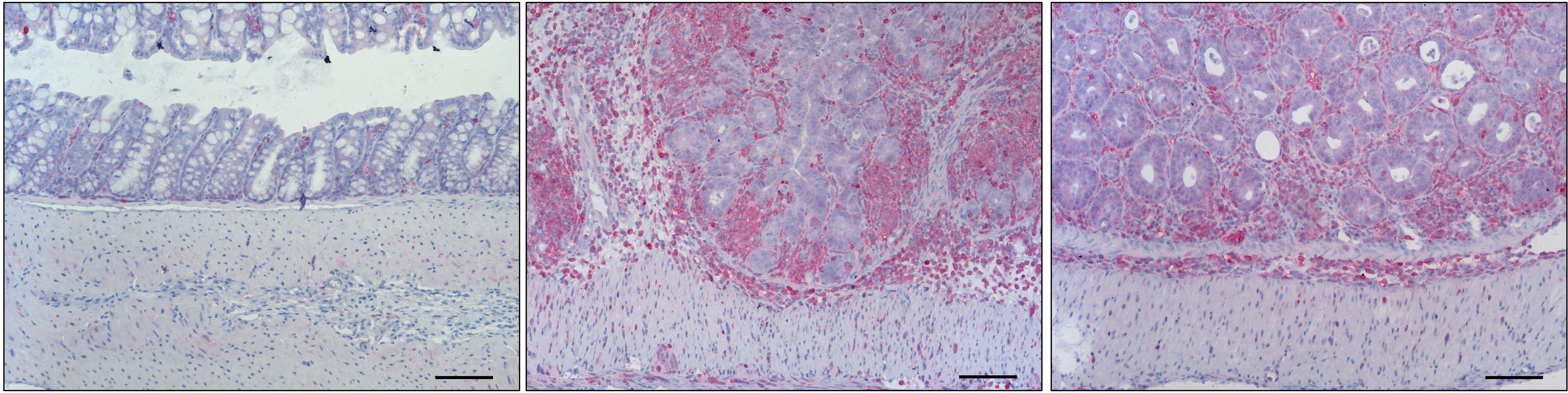

**(100 x magnification, scale bar 100 µm)**

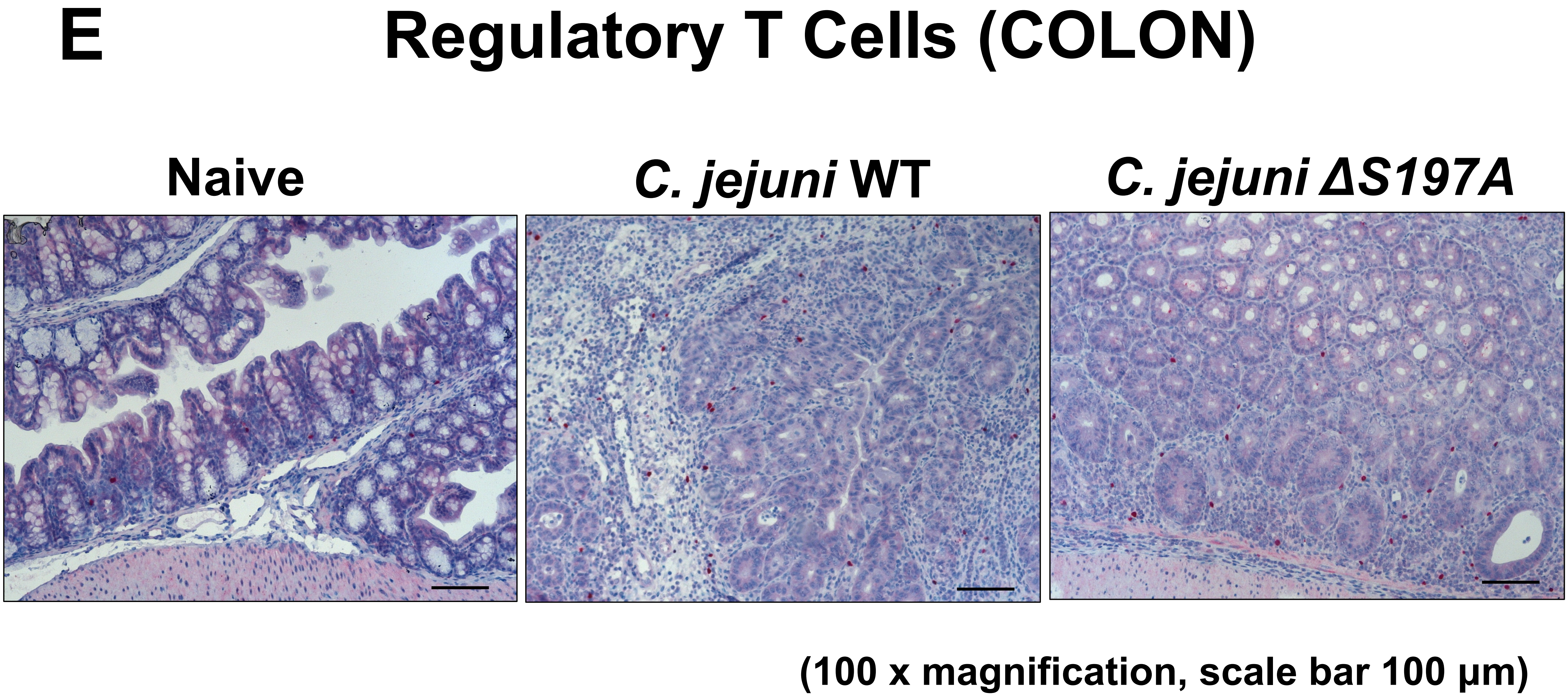
E
Regulatory T Cells (COLON)
Naive
*C. jejuni* WT
*C. jejuni ΔS197A*
(100 x magnification, scale bar 100 µm)
